# Subphrenic Abscess1Read before the Surgical Section of the Buffalo Academy of Medicine, October 3, 1899.

**Published:** 1899-11

**Authors:** Prescott LeBreton

**Affiliations:** Buffalo, N. Y.


### SUBPHRENIC ABSCESS.[1]

BY PRESCOTT LEBRETON, M. D., of Buffalo, N. Y.

AS CARL BECK wrote in 1896, subphrenic or subdiaphragmatic abscess resembles appendicitis in the fact that whereas formerly subphrenic abscess was recognised only at autopsy, now, owing to careful study of etiology and pathology, it is diagnosticated and searched for clinically. Leyden, in 1880, was the first to call the attention of the profession directly to this affection and Maydl's monograph, in 1894, summarised all the facts pertaining to the subject which then existed. Scientifically speaking, the diagnosis is the most interesting part of the subject and with this we shall chiefly deal.

Subphrenic abscess is always a complication, secondary to traumatism or disease elsewhere. There is not one abdominal organ, but has been found at sometime, when undergoing suppurative

1. Read before the Surgical Section of the Buffalo Academy of Medicine, October 3, 1899.

inflammation to have caused cases, even the pancreas. The most frequent cause is rupture of a gastric or duodenal ulcer, as Maydl found in thirty-five out of one hundred and seventy-nine cases. The second cause in order is appendicitis, the appendix usually lying behind and external to the cecum and the infection traveling upward to the diaphragm, either directly along the peritoneum or indirectly by the lymphatics. Other causes are: abscess of the kidney, perinephritis, cholecystitis, traumatism to abdomen or subphrenium, rupture of typhoid ulcers of the intestine, echinococcus cyst of the liver and pelvic abscess. To these we may add pneumonia of the lower lobe of the right lung, and empyema perforating the diaphragm.

Subphrenic abscess is most often situated on the right side in the male, owing to the fact that appendicitis and ulcer of the duodenum occur more frequently in the male sex. In females, on the other hand, there is a greater preponderance of cases of ulcer of the stomach, which frequently causes the location of the abscess on the left side.

The lesion in this disease is found to be a localised suppurative peritonitis beneath the diaphragm, either between the diaphragm and the liver or diaphragm, stomach and spleen. If the infection has been carried by the lymphatics, as from an appendix abscess, the pus may be extraperitoneal and not intraperitoneal. Experiments have shown that a colored fluid poured into the abdominal cavity is soon seen in the lymphatic vessels lining the under surface of the diaphragm. This upward course of the lymphatic current explains the complication.

As subphrenic abscess so frequently communicates with the gastro-intestinal tract, it contains, in one-third to one-half the cases, considerable gas. To this condition Leyden gave the name of "pneumothorax subphrenicus."

Lampe and Freiberg have reported cases in which there was an accompanying serous exudation in the pleural cavity. It is well to remember this, for an error in diagnosis may be made if the needle withdraws only clear serum, and a further effort should be undertaken with a longer needle to reach the pus.

A leucocytosis is regularly present, Cabot reporting four cases, in all of which there was a marked increase in the number of white cells.

Leyden's chief point in differentiating a subdiaphragmatic abscess from an empyema or pyopneumothorax was this: if in a patient with symptoms of effusion in the thoracic cavity there had never been during or at the beginning of the trouble any cough or other signs showing disease of lungs or pleura, and if there had been a previous history indicating inflammation of one of the abdominal organs, the diagnosis was probably subphrenic abscess. That this is not wholly true is well shown by Meltzer, who reported typical cases due to pneumonia of the right lower lobe. If we except, however, those rare instances, where subdiaphragmatic abscess is due to pneumonia or empyema, we have the most important clue to the diagnosis—namely, the preceding history of abdominal and not thoracic disturbance.

Pain over the lower ribs, radiating to the shoulder, is a frequent symptom, sometimes accompanied by attacks of singultus. It is of value chiefly in connection with the other signs. In subphrenic abscess we have a dome-shaped area of dulness, corresponding to the convexity of the diaphragm, beginning at the third or fourth rib in the mammary line, rising as high as the sixth rib in the axilla and then gradually descending posteriorly toward the spine. In empyema in this situation we have the opposite, the line of dulness being concave upwards. Careful percussion, mapping out the exact border, makes this test of value.

In subphrenic abscess, if the diaphragm is not yet so paralysed by pressure or by the absorption of toxins that it cannot move, a full inspiration may cause the upper line of dulness to descend and the respiratory murmur of healthy lung may be heard at a lower point. Also the external portion of the exploring needle will oscillate during respiration in the opposite direction from that of the diaphragm, the so-called Fürbringer symptom.

If the abscess is situated on the left side, it causes a dull note on percussion over the triangular space of Traube, between the precordium and the seventh rib. Only extensive cases of empyema can effect this change.

Pfuhl's method is to connect the exploring needle with a manometer and notice the change in pressure. Normally the intrathoracic pressure is greater during expiration and less during inspiration. So in pyopneumothorax the manometer shows a lessening of pressure during inspiration. In subphrenic abscess there is an increase of pressure during the inspiratory act due to the contraction of the diaphragm. If no gas is present we may simply watch the flow of liquid and not use the manometer. Several writers, however, as Carl Beck, affirm that this means of diagnosis will prove rather of theoretical than practical value.

Brown reports a case in which he aspirated pus containing the bacillus coli communis. The operation afterward proved the abscess to be subphrenic, caused by a ruptured gastric ulcer. He urges that if in any case, after aspiration, the colon bacillus is found in pure or mixed culture the pus probably lies below the diaphragm.

To differentiate a small subphrenic abscess from an abscess of the liver is very difficult. The main point here is to remember that a subphrenic abscess lifts the diaphragm more than it depresses the liver and that an abscess of the liver causes chiefly downward displacement of that organ.

The prognosis is fair when surgical intervention is early and thorough. Delay allows the pus to burrow into the pleural cavity, lungs, stomach or peritoneum, or the patient succumbs to sepsis.

The treatment is very similar to that of empyema. Resection of a part of either the eighth, ninth or tenth ribs in the middle axillary line, incision of the diaphragm, evacuation of the contents of the abscess, and drainage through one or two tubes. If the pleural cavity is encountered and is not shut off by adhesions, a circular row of sutures may be inserted and protected by gauze.

BIBLIOGRAPHY.

Maydl: Ueber Subphren. Abscesse. Wien, 1894.

Leyden: Zeitschrift für Klinische Medizin. Bd. I., p. 320.

Meltzer: On Subphrenic Abscess. N. Y. Medical Journal, June 24, 1893.

McNaught: British Medical Journal, 1897, I., 1280.

Brown: Subdiaphragmatic abscess simulating empyema. Med. and Surg. Rep. Presbyterian Hospital, N. Y., I., p. 171.

Carl Beck: Subphrenic Abscess and its relations to pyothorax. Jour. Amer. Med. Ass. Chicago, 1896. XXVI., 1050.

Freiberg: Subphrenic abscess complicating appendicitis. Cincinnati Lancet-Clinic, 1896. N. S. 37, 431.

Medical Record, 1896. 391.

Medical Record, 1896. 217.

DISCUSSION.

Dr. Park—I think emphasis should be laid on the free communication between the abdominal and pleural cavities by means of the lymphatic vessels in considering the etiology of this affection.

Dr. Lyon—Quoting from Cantlie's monograph on this subject laid especial stress on the value of blood examination in this condition. Bacteriological examinations from pus found in these abscesses were frequently found to be sterile. He thinks the amebæ coli is the most frequent cause in this affection.

Dr. Krauss—Reported that a pupil in Ehrlich's clinic stated that the presence of a leucocytosis in deep seated abscess does not show anything characteristic and questions the utility of blood examinations in this affection.

Dr. Hartwig—In discussing the operative procedure does not think that if the diagnosis has been positively made between a subphrenic and intrathoracic abscess that going through the pleural cavity is to be recommended, even if the pleural surfaces are stitched together before entering the abscess. He advises opening anteriorly or posteriorly through the peritoneal cavity, as the case may indicate, though transpleural successes are recorded.

